# Reproductive health service utilization and associated factors among secondary school students in Harari regional state, eastern Ethiopia, 2022: a multicenter study

**DOI:** 10.1186/s12978-023-01592-1

**Published:** 2023-03-17

**Authors:** Addisu Sertsu, Addis Eyeberu, Tilahun Bete, Elias Yadeta, Magarsa Lami, Tegenu Balcha, Bekelu Berhanu, Ayichew Alemu, Fentahun Meseret, Hanan Mohammed, Addisu Alemu, Ahmed Mohammed Husen, Fila Ahemed, Abdi Birhanu, Kabtamu Gemechu, Adera Debella, Tamirat Getachew, Kabtamu Nigussie, Shambel Nigussie, Abraham Negash

**Affiliations:** 1 School of Nursing and Midwifery, College of Health and Medical Sciences, Haramaya University, Harar, Ethiopia; 2 School of Public Health, College of Health and Medical Sciences, Haramaya University, Harar, Ethiopia; 3 School of Medicine, College of Health and Medical Sciences, Haramaya University, Harar, Ethiopia; 4 School of Laboratory Sciences, College of Health and Medical Sciences, Haramaya University, Harar, Ethiopia; 5 School of Pharmacy College of Health and Medical Sciences, Haramaya University, Harar, Ethiopia

**Keywords:** Reproductive health, Service utilization, Secondary school, Harar, Ethiopia

## Abstract

**Introduction:**

Reproductive health encompasses all conditions relating to the reproductive system and goes beyond simply being free from disease or infirmity. Several socioeconomic and socio-cultural factors affect reproductive health service utilization.

**Objectives:**

To assess reproductive health service utilization and its associated factors among government secondary school students in Harari regional state, Eastern Ethiopia 2022.

**Methods:**

A school-based cross-sectional study design was conducted among 1275 secondary school students in six randomly selected secondary schools in Harari Regional state, in eastern Ethiopia. The study participants were chosen using a simple random sampling method. Data was gathered using self-administered questionnaires, entered into Epi Data version 3.1, and exported to SPSS version 25 for cleaning and analysis. Descriptive statistics, bivariable, and multivariable logistic regression analyses were carried out to compute the frequency of each independent variable and the magnitude of the outcome variables, then to identify factors associated with the outcome variable, respectively. To declare a significant association, an adjusted odd ratio (AOR) with a 95% confidence interval and a p-value of 0.05 were used.

**Results:**

Our finding indicated that 25.3% (95% CI:22.9, 27.7) of the secondary school students utilized reproductive health services. Being in grade 11–12 (AOR = 1.67, 95% CI: 1.18, 2.38), having a history of sexually transmitted infection (AOR = 6.11, 95% CI: 2.20, 16.99), presence of a health facility nearby (AOR = 1.49, 95% CI: 1.12, 1.99), discuss voluntary counseling and testing with family (AOR = 2.73, 95% CI: 1.90, 3.94), and discussing about contraceptive with friends (AOR = 1.22, 95% CI: 0.91, 1.65) were the elements that had a strong correlation with reproductive health service utilization.

**Conclusion:**

In this study, only one-fourth of secondary school students utilized RH service during the past year. The student's educational level, having a history of STI, the presence of a health facility nearby, and discussing RH service with family/friends were the factors significantly associated with reproductive service utilization among secondary school students.

## Introduction

Reproductive health encompasses all conditions relating to the reproductive system's physical, mental, and social well-being and goes beyond simply being free from disease or infirmity. This indicates that individuals can reproduce and have the flexibility to decide whether, when, and how often to do so [1, 2]. According to the World Health Organization, adolescents are young people between the ages of 10 and 19 [3, 4]. One of life's most rapid and complex stages, marked by significant changes in the physical, cognitive, behavioral, social, and psychological domains, occurs during this age [5]. At this young age, they are vulnerable to risk and unintentional harm due to poor decisions and actions [6].

Around 1.2 billion adolescents are alive today, and more than half of them live in developing countries [7]. 33.8% of Ethiopia's population is between the age of 10 to 24; 22% of this group are adolescents [8, 9]. Although young people are regarded as a country's best hope for the future, their immaturity exposes them to unique risks such as unwanted pregnancy, STIs such as HIV, and unsafe abortion [10, 11].

Secondary school adolescents are particularly at risk for RH problems since they frequently engage in risky sexual activity [12]. The use of reproductive health services has been linked to several socio-demographic and socio-economic factors, including age, being a female adolescent, and parental communication. Maternal education, religious activities, maternal education, and media exposure are also important factors in the uptake of these services [13]. Utilization of SRH services varies widely across the country, ranging from 29.4% Hadiya zone South Ethiopia [14] to 63.8% in Harar [15]. Some evidence indicated that high school students were avoiding SRH facilities due to the unfavorable service hours, anxiety about being seen by others, long waiting times, and unwelcoming and judgmental staff members [16]. Even though it is not a regular event, schools are the primary source of information on reproductive health issues [17].

The Ethiopian government, in collaboration with several non-governmental organizations, has been actively encouraging programs including the institutionalization and scaling up of youth-friendly services [9, 18]. The results of all the initiatives, however, have not been seen in Ethiopian educational institutions, as evidenced by the enduring problems with young people's reproductive health. For example, the prevalence of STIs, including HIV/AIDS (19.5%), is increasing, and abortion rates among students are 65 per 1,000 women, which is three times the national average for Ethiopia [12, 19].

In the study area and the country as a whole, there is a scarcity of recent evidence on the use of RH services by secondary school students. Therefore, the goal of this study was to assess secondary school students in the Harari Regional State of eastern Ethiopia for their use of RH services and related features.

## Methods

### Study setting and period

The study was carried out in the selected secondary schools in the Harari regional state from April 10 to May 10, 2022. One of Ethiopia's ten regions, Harari Regional State is found in the country's eastern area, 525 km from Addis Ababa, the country's capital. Nine woredas, three rural and six urban make up the region. The urban districts are subdivided into 19 kebeles, and the rural districts are subdivided into 17 peasant associations (which is equivalent to kebeles in the urban case).

The region has a total population of 270,00, of which 136,000 are men and 134,000 are women (2021 projection based on the 2007 Census, CSA). There are ten govt secondary schools in the area, and in 2022, 7000 students were enrolled there. 43.3% of those students were female.

### Study design and source population

An Institutional based multicenter cross-sectional study was conducted. In this study, the source populations were all secondary school students in Harari Regional State, and students in selected secondary schools available during the data collection period were included, whereas critically ill students were excluded from the study.

### Sample size determination and sampling procedure

The required sample size for this study was calculated using a single population proportion formula (n = (Z/2)2 p(1-p)/d2)); where n: is the required sample size, Za/2 (1.96): is the significance level = 0.05 at 95% confidence interval design effect = 1.5, the margin of error (d): 0.03, and p: is the proportion of reproductive health utilization among high school students in south Gonder, northwest Ethiopia, 24.6% [20]. After adding a 10% non-response rate, 1306 was the final calculated sample size for this study.

**Fig. 1:**
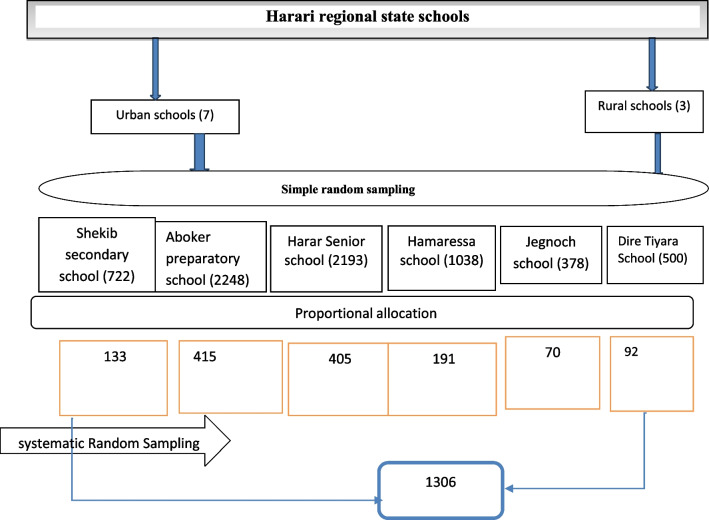
Schematic presentation of sampling procedure for selection of study participants among secondary students in Harari regional sate, Eastern Ethiopia, 2021

The study participants were chosen using a multiple-stage sampling procedure. The regional state of Harari had ten high schools. Six secondary schools were chosen at random after the schools were stratified into rural and urban areas. Then, to choose a representative sample size from each secondary school, the total number of students in each chosen school was obtained, and a proportional allocation was done. The student registration book was utilized as a sampling frame for systematic random sampling (Fig. 1).

### Data collection tools and procedures

A self-administered questionnaire was used to collect the data. A pretested and structured questionnaire was adopted by reviewing different literature [21–24]. Then the questionnaire was modified for the local context and used to collect data about reproductive health services utilization. The data was collected by 15 well-trained BSc nurses and supervised by eight MSc nurses. A brief introductory orientation was given to the study participants by the data collectors about the purposes of the study. The significance of their participation was explained, and then volunteers were interviewed for the study.

### Measurement and operational definitions

Reproductive health service utilization is the outcome variable of the study. It was measured as a composite variable if there is the utilization of at least one of its components. The following questions were asked with yes or no responses. Do you get an STI diagnosis and treatment? Do you get contraceptive services? Ever used a voluntary counseling test? Have you ever used perinatal service? Have you ever used an abortion service?

#### Utilization of reproductive health services

Utilization of any one of the following RH services: contraceptive service, perinatal service, voluntary counseling and testing (VCT), sexually transmitted infections (STI): screening, diagnosis, and management, and abortion services [25].

#### Health facility availability

Measure the distance to the facility as an interval categorical variable with two categories: less than 2 km to a facility, and greater than 2 km to a facility [26].

### Data quality control

The questionnaire was initially prepared in English and then translated into the local languages by a language expert. Then, it was translated back into an English version to ensure its consistency. A pre-test was done on 5% of the total sample size at Haramaya on a similar population. Based on expert comments and pretest finding a necessary amendment was made to the questionnaire to ensure its consistency before the actual collection of data. The goal of the study, sampling process, components of the questionnaire confidentiality and privacy are all covered in training provided for data collectors and supervisors. Daily supervision was done, and data were checked for completeness, correctness, and clarity before entry.

### Data processing and analysis

The collected data were coded, cleaned, and fed into Epi-data version 3.1 and analyzed using SPSS version 25 software. Descriptive statistics (frequency, percentage, mean, median, and interquartile range) were used to describe the characteristics of participants and presented using tables, pie charts, and bar graphs. Bivariable and multivariable logistic regression analysis was done to identify factors associated with reproductive health services utilization. Multicollinearity was cheeked by variance inflation factor and tolerance test, model fitness was also cheeked by the Hosmer–Lemeshow test and the model was well fitted. Variables with -value ≤ 0.2 in the Bivariable logistic regression were included in the multivariable logistic regression. Association was described using an adjusted odds ratio along with a 95% confidence interval and a p-value < 0.05 was considered to declare a statistically significant association.

## Results

### Socio-demographic characteristics

A total of 1306 secondary school students were interviewed, with a 97.6% response rate. The median age of the respondents was 17, with an interquartile range of 16–18 and ranging from 13 to 30 years. Among the study participants, 982 (77%) were single, and more than half (54.3%) of the participants were girls. 835 (65.5%) respondents were Muslim, and 346 (27.1%) were orthodox Christian followers. In total, 82.9% of study participants live with their families, while 38 (3%) live alone (Table 1).

**Table 1:** Socio-demographic characteristics of high school students in Harari region, Ethiopia, 2022

Variable	Category	Frequency	Percentage (%)
Age	$\le 18$	1015	79.6
	$>18$	260	20.4
Educational status	9–10	1053	82.6
	11–12	222	17.4
Religion	Orthodox	346	27.1
	Muslim	835	65.5
	Protestant	81	6.4
	Catholic	8	0.6
	Others	5	0.4
Marital Status	Single	982	77.0
	In relationship	250	19.6
	Married	43	3.4
Resident	Rural	339	26.6
	Urban	936	73.4
With whom do you live?	With parents	1057	82.9
	With nonparents	180	14.1
	Alone	38	3.0
Mother education level	Have no formal education	422	33.1
	Elementary	503	39.5
	Secondary	195	15.3
	Diploma and above	155	12.2
Father education level	Have no formal education	245	19.2
	Elementary	352	27.6
	Secondary	373	29.3
	Diploma and above	305	23.9
Do you get pocket money	Yes	845	66.3
	No	430	33.7
Does your family control you	Yes	909	71.3
	No	366	28.7

### Study participants' sexual practices and reproductive health service utilization

127 participants (about 10%) reported engaging in sexual activity. 61 (4.8%) of the total study participants used condoms during their most recent sexual activity. 188 (14.1%) had used youth-friendly services in the last year. In the previous year, forty (3.1%) of secondary school students became pregnant. Utilization of reproductive health services was 25.3% among the respondents. The most utilized aspects of reproductive health services were voluntary counseling and testing and the use of contraceptives, which accounted for 14.1% and 10.1%, respectively (Table 2).

**Table 2:** Information and sexual and RHSUamong secondary school students in Harari Region, Ethiopia, 2022

Variable	Category	Frequency	Percentage (%)
Have you had sex in your life	Yes	127	10
	No	1148	90
Have you make sex in your last year	Yes	118	9.3
	No	1157	90.7
Have you used a condom in your last sex	Yes	61	4.8
	No	1214	95.2
Sexual Victim	Yes	35	5.1
	No	1240	94.9
Have you ever discussed family planning with your friends	Yes	328	25.7
	No	947	74.3
Have you ever used any method of family planning	Yes	107	8.4
	No	1168	91.6
Is there a health facility nearby	Yes	831	65.2
	No	444	34.8
Have you ever pregnant	Yes	40	3.1
	No	1235	96.9
Ever used YFS	Yes	188	14.7
	No	1087	85.3
Discussed with parents On VCT	Yes	156	12.2
	No	1119	87.8
Do you get STI diagnosis and treatment	Yes	20	1.6
	No	1255	98.2
Do you get contraceptive service	Yes	132	10.4
	No	1143	89.6
Ever used a voluntary counseling test	Yes	193	15.1
	No	1082	84.9
Have you ever used perinatal service	Yes	27	2.1
	No	1248	97.9
Have you ever used an abortion service	Yes	66	5.2
	No	1209	94.8
RHS Utilization	Yes	322	25.3
	No	953	74.7

### Factors associated with reproductive health service utilization

Age, marital status, having sex in the last year, being a victim of sexual abuse, being diagnosed and treated with STI, the presence of a health facility nearby, discussing voluntary counseling and testing with family, and discussing contraception with friends all exhibited p-values less than 0.2 in bivariate logistic regression analysis.

In the bivariable logistic regression analysis, variables with a P-value of less than 0.2 were chosen for the multivariable logistic regression analysis to find variables with a p-value of less than 0.05 that were significantly associated with the use of reproductive health services.

However, only educational level, having been diagnosed and treated for STI in the past, discussing RHS with family and friends, and living near a health facility were significantly associated with reproductive health service utilization in multivariable logistic regression analysis.

Students who are in grades 11–12 were 1.65 times (AOR = 1.65, 95% CI: 1.14, 2.31) more likely to utilize reproductive health services than those who were in 9–10 grades. Students who had STI diagnosis and treatment were 6.68 times more likely to use the service (AOR = 6.68, 95% CI: 2.41, 18.55). When compared to individuals who lived far from a health facility, those who were close to one had a 1.41 (AOR = 1.41, 95% CI: 1.05, 1.88) times higher likelihood of using reproductive health services. Discussing and communicating about reproductive health services with family and friends has a 2.62 (AOR = 2.62, 95% CI: 1.82, 3.79) times higher likelihood of using reproductive health services than those who didn't communicate about the issue (Table 3).

**Table 3:** Factors associated with RHSU among high school students in Harari Regional State, 2022

Variables	RHS utilization		COR (95% CI)	AOR (95% CI)	p-value
	Yes	No			
*Age of participant*					
$\le$ 18 years	236	779	1	1	
> 18 years	86	174	1.63 (1.21, 2.19)	1.32 (0.93, 1.86)	0.11
*Marital status*					
Single	240	742	1	1	
In relationship	63	187	1.04 (0.76, 1.43)	0.94 (0.67, 1.33)	0.76
Married	19	24	2.44 (1.32, 4.54)	1.50(0.75, 3.00)	0.24
*Educational status*					
9–10	245	808	1	1	1
11–12	77	145	1.75 (1.28, 2.39)	1.67 (1.18, 2.38)	0.00
*Have you make sex in the last* year					
Yes	49	69	2.3 (1.55, 3.70)	1.40 (0.83,2.36)	0.20
No	273	884	1	1	
*Faced Victim of sexual abuse*					
Yes	15	20	2.28 (1.15, 4.50)	1.12( 0.47, 2.68)	0.79
No	307	933	1	1	
*History of STI*					
Yes	14	6	7.17 (2.73, 18.83)	6.11 (2.20, 16.99)	0.00
No	308	947	1		
*Health facility nearby*					
Yes	233	598	1.32 (1.55, 1.17)	1.49 (1.12, 1.99)	0.00
No	89	355	1		
*Discussed VCT with family*					
Yes	71	85	2.88 (2.04,4.02)	2.73(1.90, 3.94)	0.00
No	251	868	1	1	
*Discussed family planning*					
Yes	103	225	1.52 (1.15, 2.09)	1.22 (0.91, 1.65)	0.17
No	219	729	1	1	

AOR: adjusted odd ratio, COR: crude odd ratio, PV: p-value

## Discussion

The findings of this study revealed that 25.3% (95%CI; 22.9–27.5%) of high school students utilized RH services in the past year. The findings of this study were consistent with those of studies carried out in Gonder, Northern Ethiopia, where the results were 24.6% [27], and Haramaya district, Eastern Ethiopia, where the results were 23.5% [28]. This study's findings, however, were lower than those of Adama (34%) [29], Amhara region 54.6% [30], Debra tabor, northwest Ethiopia, 28.8% [31], Woldia northern Ethiopian, 64.3% [32], and Nigeria 51% [33]. The reason behind this might be due to the large sample size utilized in the current study. Furthermore, the Amhara region study only assessed girls, indicating that many biological, socioeconomic, and sociocultural factors increase the vulnerability of girls to RH problems, and as a result, their rate of utilizing the service may be higher than that of males.

The study result was higher than the study conducted in Nekemte, Western Ethiopia, at 21.2% [25], Mecha district northwest Ethiopia, at 18% [34], and Nepal 9.2% [35]. The reason for this was, methodological and study setting deference. Participants' sociodemographic information and cultural influences, the distance of the facility, newly generated, and improvement in health information dissemination also contributed to this discrepancy.

According to our research findings, those who were in grades 11 and 12 were 1.6 times more likely to use RHS than grade 9 and 10 students. The study done in Adama [29], Gondar [27], and a systematic review and meta-analysis study conducted in Ethiopia [36] support this finding. The possible explanation for this might be that those whose grade levels were 11–12 had more information access about RHS utilization as compared to their counterparts, and secondary sexual characteristics also increased as the grade level was increased.

Those who had a history of STI were 6.1 times more likely to use RHS than those who did not have STI. The Nekmte study was used to support the report [25]. This may be justified by the fact that people with a history of STIs may go to a medical facility and will get STI treatment and other components of RHS.

Students who live in a nearby health facility were 1.4 times more likely to utilize RHS than those who live at a far distance from the health facility. Other studies conducted in Ethiopia and abroad also reported similar findings; Adama [29], Gonder [27], a systematic review and meta-analysis study in Ethiopia [36], and Nepal [35]. However, the Haramaya district eastern Ethiopia study was used to contradict this finding [28]. Location benefits could be the cause of this. Those who were at close-by facilities may have adequate awareness of the service that was available and can be used easily.

Those participants who discussed voluntary counseling and testing with their families were 2.7 times more likely to utilize RHS than those who didn't. The finding was in line with a systematic review and meta-analysis study in Ethiopia [36], a study done in the west Arsi zone [37], and a comparative study conducted in southern Ethiopia [38]. The possible explanation for this was that those who discuss reproductive health problems openly with their family and friends know the health impacts of the RH problem more than their counterparts, they have no fear of using the service, and they might also obtain family support to visit the RH clinic.

### Strengths and limitations of the study

This study has some restrictions despite making use of sizable sample size and pretested tools. Due to the sensitive nature of the subject under the study, respondents might not be completely honest while responding, this might understate the outcome of the use of RH services.

## Conclusion

Only one-fourth of secondary school students used RH services in the previous year, according to this study. The educational level of the student, having a history of STI, distance from the health facility, discussing RH service with family/friends, and perceiving the risk of pregnancy were the variables significantly associated with reproductive service utilization among secondary school students. Considering regular health education about reproductive health at school is very important to improve service utilization.

## Data Availability

All supplemental materials for this article is available from the corresponding authors based on reasonable request.

## Abbreviations

RHSU: Reproductive health service utilization
STI: Sexually transmitted infection
VCT: Voluntary counseling and testing
YFS: Youth-friendly service
